# Effects of Moderate Aerobic Exercise on Cognitive Abilities and Redox State Biomarkers in Older Adults

**DOI:** 10.1155/2016/2545168

**Published:** 2016-04-18

**Authors:** Ahmad H. Alghadir, Sami A. Gabr, Einas S. Al-Eisa

**Affiliations:** ^1^Department of Rehabilitation Sciences, College of Applied Medical Sciences, King Saud University, Riyadh 11433, Saudi Arabia; ^2^Department of Anatomy, Faculty of Medicine, Mansoura University, Mansoura 35516, Egypt

## Abstract

We used a moderate aerobic exercise program for 24 weeks to measure the positive impact of physical activity on oxidative stress and inflammatory markers and its association with cognitive performance in healthy older adults. A total of 100 healthy subjects (65–95 Yrs) were randomly classified into two groups: control group (*n* = 50) and exercise group (*n* = 50). Cognitive functioning, physical activity score, MDA, 8-OHdG, TAC, and hs-CRP were assessed using LOTCA battery, prevalidated PA questionnaire, and immunoassay techniques. LOTCA 7-set scores of cognitive performance showed a significant correlation with physical activity status and the regulation of both oxidative stress free radicals and inflammatory markers in all older subjects following 24 weeks of moderate exercise. Physically active persons showed a higher cognitive performance along with reduction in the levels of MDA, 8-OHdG, and hs-CRP and increase in TAC activity compared with sedentary participants. Cognitive performance correlated positively with the increase in TAC activity and physical fitness scores and negatively with MDA, 8-OHdG, and hs-CRP, respectively. There was a significant improvement in motor praxis, vasomotor organization, thinking operations, and attention and concentration among older adults. In conclusion, moderate aerobic training for 24 weeks has a positive significant effect in improving cognitive functions via modulating redox and inflammatory status of older adults.

## 1. Introduction

Cognitive abilities refer to all essential mental skills that control the behavioral lifestyle of humans such as everyday routine work [1]. Decline in cognitive abilities was shown to produce more drastic problems for older adults in performing their daily life activities [2, 3]. However, more studies tried to maintain or enhance cognitive abilities in older adults via enhancing or delaying functional disabilities [4]. The results of these trials are not clear; this is maybe due to the fact that most of these trials concentrated on treatment schedules rather than prevention in older subjects with cognitive deficits or functional disabilities.

Previously it was reported that prevention or improvement of cognitive deficits among normal older adults is accessible but the treatment parameters did not include the outcome measures which may be related to the limited sample randomization and lack of it [5, 6].

The impairment in brain function in older age occurs via many pathological mechanisms [7–10], the most important of which are tissue damage and neural cell death which occurs via the interaction of complex pathophysiological processes [11].

It was reported in many studies that human aging is demarcated with an increase in serious age-related diseases, which related to chronic pathological processes such as inflammation. Whereas the incidence of inflammation along with immunosenescence results in a decline of multiple physiological systems and functional dependence among the elderly [12–14], the negative effects of the imbalance between pro- and anti-inflammatory cytokines on cognitive abilities such as memory and learning deficits were greatly reported among older people with Alzheimer's disease [15].

Many research studies reported that C-reactive protein (CRP) and high-sensitivity CRP (hs-CRP) markers of chronic inflammation have been associated with numerous clinical conditions, including cognitive decline and depression in old age [16–21]; the causative effects of higher CRP or hs-CRP inflammatory markers on cognitive impairment speculatively occurred via promoting vascular disease or as a result of the inflammatory process linked with disorders in many lifestyle factors including obesity, physical inability, and smoking [22].

Also, many research studies supported the pathogenic role of oxidative stress in chronic diseases related to human aging like cognitive impairment. It is widely accepted that oxidative stress is characterized by disturbance in the hemostatic balance between oxidative stress free radicals such as reactive oxygen species (ROS) and reactive nitrogen species (RNS) in human cells and the ability of these cells to conquer this change by their own antioxidant defense pathways [23, 24]. It was reported that older people with cognitive disorders showed lower activity in antioxidant pathways like antioxidant enzymes (catalase, superoxide dismutase, and glutathione peroxidase) and numerous nonenzymatic antioxidants (GSH, vitamins A, C, and E, and carotenoids) [10, 11, 25–27]. The increase in oxidative free radicals leads to their higher accumulation in human neural cells ultimately damaging lipids, proteins, and DNA [28, 29] and produces more biologically active molecules such as malondialdehyde (MDA) and 8-hydroxyguanine (8-OHdG) adducts which are liberated from oxidative cellular damage of the polyunsaturated fatty acids and DNA, respectively. These newly formed free radicals may be involved in further oxidation reactions, generating new oxidative damage [30, 31], and that may be recognized as a reason for brain disorders, cognitive decline, and dementia in old age [32].

Many studies focused on the importance of body physical activity and its positive effects upon cognitive abilities especially in older ages. Physical exercise was shown to play a protective role against hippocampal cell injury which produces brain memory loss [33–36]. Also, physical activity facilitates recovery from injury and improves cognitive function via increase of the expression of many neurotrophic and physiological factors involved in neural survival, differentiation, and improvement of memory function [37–41].

Recent studies reported the potential action of exercise as an antiapoptotic parameter against many brain diseases such as brain inflammatory conditions [42] and mice Parkinson's disease [43] and in the improvement of depressive symptoms [44] and traumatic brain injury [45, 46] and alleviation of memory impairment [47].

It was reported that physical exercise exerts good effects on cognitive abilities with different ways which argue its importance as nondrug and noninvasive essential targets for long term health programs for all ages [48, 49]. The marked improvement was manifested on both function and biomarker integrity as shown in recent studies [50, 51]. Previously, it was reported that antiapoptotic effect of exercise depends mainly on modulation of many physiological processes including DNA damage, oxidative stress, and hormonal changes which are involved in the regulation of apoptosis in various cell types [52]. This depends mainly upon the type of exercise, its intensity, frequency, and duration as the training endpoint [53, 54]. Thus, the benefits of regular physical exercise as a health-ensuring necessity over age, gender, occupation, and affective status cannot be overestimated [55, 56].

Therefore, the present study was designed to evaluate the effects of 24 weeks of moderate aerobic exercise on the levels of oxidative stress, MDA, 8-OHdG, TAC, and hs-CRP inflammatory markers and its association with cognitive performance in healthy older adults.

## 2. Material and Methods

### 2.1. Subjects

The participants involved in this study were subjected to randomized selection. A random selection of 200 subjects on electoral roll was informed for participation. Out of them, only 100 healthy subjects (70 males, 30 females) were randomized into this study. Their age ranged between 65 and 95 years and mean age was 69.7 ± 5.91 (Table 1). Subjects with physical disability and with endocrine, immune, and psychiatric illness and eating disorders and taking glucocorticoid medication that could interfere with apoptotic and cognitive ability measurements were excluded from this study. Based upon participation in exercise program, subjects were classified randomly into two groups: control group (*n* = 50) and exercise group (*n* = 50). Demographic and anthropometric data of participants were included in Table 1. This study was approved by the Ethical Committee of the Rehabilitation Research Chair of King Saud University (file ID: RRC-2012-08).

#### 2.1.1. Training Procedure

Participants were involved in exercise program designed according to Karvonen's formula [57], three times per week for 24 weeks, whereas training intensity of each intervention was prepared according to maximum and resting heart rate of each participant. During warming the subject performed stretching exercises and walking for 5 to 10 minutes. During the active phase, the subject was allowed to reach his precalculated training heart rate (THR max: 60 to 70% for 45–60 min) in bouts form using treadmill, bicycle, and StairMaster [58, 59]. The exact calculated heart rate of each participant was monitored via a wearable automatic portable heart rate meter (Polar Electro, Kempele, Finland). The exercise test was performed to give the participants physical activities corresponding to 30–45% of VO_2_ max uptake [60].

#### 2.1.2. Leisure-Time Physical Activity (LTPA)

A validated questionnaire was used to calculate physical activity in the form of a leisure-time physical activity (LTPA). The energy expenditure rates were calculated weekly in metabolic equivalents per hour/week (T-LPTA-MET/H/W) as previously reported [61].

*Assessment of Cognitive Abilities*

*Instrument.* Trained research assistants assessed the cognitive abilities of older adults before and after supervised aerobic exercise using the Loewenstein Occupational Therapy Cognitive Assessment (LOTCA) battery. Assessments required between 45 and 90 minutes. The LOTCA consists of seven major domains divided into 26 subtests, with each subtest scored on a four- or five-point Likert scale. The assessment of LOTCA test was performed according to instruction manuals as reported in literature [62].

Results are presented as a profile along all subtests. A composite score for each domain was calculated by summing the scores of the relevant subtests. The LOTCA score was calculated by summing the scores of all subtests. The maximum score on the test is 123, and the minimum score is 27. A higher score indicates better cognitive performance.

*LOTCA Test Validity.* The test has excellent intrarater reliability (100%) and good interrater reliability (86%) as well as criterion validity (78%) [63]. This LOTCA test was chosen because of its psychometric properties and primarily nonverbal nature, making it potentially more suitable for evaluating the cognitive abilities of individuals from non-Western and non-English-speaking cultures. Several studies have been conducted using this instrument in both Western [63] and Arab populations [1]. 

*Assessment of Oxidative Stress and Inflammatory Parameters.* All serum samples were taken from all participants in the morning following an overnight fast at pre- and postexercise training program estimation of the following parameters.

*Analysis of hs-CRP.* The acute-phase reactant highly sensitive CRP (hs-CRP) is analyzed using commercially available ELISA kits (IBL Inc., Cat. Number: IB59126, USA) according to manufacturers' instructions.

#### 2.1.3. Total Antioxidant Capacity (TAC)

Serum total antioxidant capacity (TAC) was measured by Colorimetric Assay Kit (Catalog #K274-100; BioVision Incorporated, CA 95035, USA). The antioxidant equivalent concentrations were measured at 570 nm as a function of Trolox concentration according to the manufacturer's instructions:

(1) $$\begin{array}{c} \frac{Sa}{Sv}=nmol/\mu l\;\;or\;\;mM\;\;Trolox\;\;equivalent, \end{array}$$

where Sa is the sample amount (in nmol) read from the standard curve; Sv is the undiluted sample volume added to the wells. 

*Estimation of Malondialdehyde (MDA) and 8-Hydroxyguanine (8-OHdG).* Lipid peroxidation was estimated quantitatively by analyzing the levels of malondialdehyde using high performance liquid chromatography as reported previously in the literature [64]. Immunoassay technique was performed to estimate serum 8-OHdG as a marker of DNA damage using a commercially available ELISA kit (DNA Damage ELISA Kit, Product #: EKS-350, Stressgen Co., USA).

### 2.2. Statistical Analysis

Statistical analysis was performed using SPSS version 17. The data were expressed as mean ± SD. The comparison and correlation of the studied parameters were investigated using both Student's *t*-test and Pearson's correlation coefficient, respectively. The data was deemed to be significant at *P* values <0.05.

## 3. Results 

A total of 100 healthy subjects were involved in this study. Seventy percent of the sample was male (*n* = 65), and 60% of subjects were highly educated (*n* = 60). They are classified according to exercise program into control group (*n* = 50) and exercise group (*n* = 50). There was significant difference in WHR (*P* = 0.05), LPTA (*P* = 0.001), and average LOTCA scores (*P* = 0.01) of exercise participants compared to control group (Table 1).

In this study, there was a significant (*P* = 0.01) improvement in all LOTCA 7-subset variables among subjects following 12 weeks of moderate aerobic training compared to pretest and control group that showed slight improvement in LOTCA scores as shown in Table 2. However, significant increase (*P* = 0.001) in the improvement of motor praxis, vasomotor organization, thinking operations, and attention and concentration was reported among older adults following 24-week aerobic exercise. The data revealed positive significant correlations between the LOTCA scores of older subjects and their performance of cognitive abilities. Moreover, significant correlations were obtained between the older subjects in the motor praxis, vasomotor organization, thinking operations, and attention and concentration domains of the LOTCA scores and their performance of functional physical activity as shown in Table 5.

Oxidative stress and inflammatory makers TAC, MDA, 8-OHdG, and hs-CRP were greatly reported in this study. There was significant increase in the activity of TAC and decrease in the levels of MDA, 8-OHdG, and hs-CRP in participants of exercise group following 24 weeks of moderate aerobic training. Also, significant increase in LPTA activity was observed in exercise group compared to pretest and control group as shown in Table 3.

Also, the data showed significant correlation between oxidative stress markers and the levels of hs-CRP inflammatory markers. There was significant positive correlation between MDA and 8-OHdG along with negative correlation of TAC activity in both participants of normal and abnormal hs-CRP. The data obtained proposed significant interrelations between oxidative stress free radicals which may produce inflammation resulting in increase in hs-CRP levels (Table 4).

The changes in these parameters were shown to be significantly correlated with physical fitness score. In physically active participants, the physical fitness score correlated negatively with the reduction in the levels of MDA, 8-OHdG, and hs-CRP markers and positively with the increase in TAC activity compared to those of lower activity scores (Table 5). Physically active persons showed higher cognitive performance with lower expression in the levels of MDA, 8-OHdG, and hs-CRP and significant increase in TAC activity compared to less active ones. The improvements of cognitive performance of physically active persons correlated positively with the increase in TAC activity and negatively with other stress and inflammatory markers, MDA, 8-OHdG, and hs-CRP, respectively (Table 6).

## 4. Discussions

Physical activity as a nondrug modulation is considered one of the most promising strategies to prevent or improve cognitive disabilities among elderly populations [65]. It was reported that physically active people across their entire life minimize the incidence rates of dementia and cognitive difficulties [66–68], whereas many studies reported lower rates of cognitive disorders among subjects who participated in higher levels of physical activity interventions than persons with lower scores of physical activity [69, 70]. Recently, many research works revealed that physical exercise with moderate intensity produces remarkable higher levels of improvement in skills, mobility, and mood in both younger [71] and older [72] adults. However little is known about the positive effect of moderate exercise on cognitive abilities which occurs via modulating oxidative stress and inflammation profile.

Therefore, the current research work aimed to investigate the probable correlation between antioxidative and anti-inflammatory mechanisms of exercise on cognitive abilities among 100 older adults that participated in supervised aerobic training program for 24 weeks.

In the present study, the cognitive abilities of 100 older adults of both control and exercise group were measured using LOTCA scores, a cognitive evaluation test formed of 7-subset variables. In older subjects that participated in moderate aerobic exercise for 24 weeks, there was significant improvement in cognitive performance via increase in all LOTCA 7-subset variables compared to nonexercised group. The data revealed positive significant correlations between the total LOTCA scores of older subjects and their performance of cognitive abilities. Thus, the accuracy and evaluation of LOTCA test support its use as a diagnostic tool for cognitive function as previously reported [1].

Moreover, significant positive correlation was obtained between the older subjects in the motor praxis, vasomotor organization, thinking operations, and attention and concentration domains of the LOTCA scores and their performance of functional physical activity. The data matched with others who suggested the strongest indication of physical exercise benefits on cognition function via enhancing academic performance and psychological well-being [40, 73].

Similarly, our study was in accordance with recent studies that reported improving in cognitive performance on a working memory task among younger and older adults following moderate intensity cycling [74]. Also, our study supported that positive effects of physical exercise intervention are relayed in enhancing psychological well-being, cognitive functioning, and quality of life especially in older subjects with mild cognitive impairment as reported recently in literature [39, 75].

In the present study, there was slight insignificant change in cognitive abilities scores and oxidative and inflammatory related markers among nonexercised control group. This change may be due to low-intensity physical activity such as routine daytime life which accounts for most activity energy expenditure (AEE) in people who do not regularly exercise [76]; these activities may be useful in health outcomes such as cognitive impairment. This is indicated with other research work that reported that older women identified a positive association between cognitive performance and total daytime movement which suggests that total activity may be important for cognitive outcomes [77].

Cognitive disabilities among older adults occurred as a result of brain dysfunction which occurs through many pathological mechanisms including brain tissue damage and neural cell death or apoptosis induced by oxidative free radical and inflammation mechanisms [7, 8, 27, 78].

In this current study, the anti-inflammatory activity of moderate exercise was evaluated in older adults following 24-week exercise interventions. There was decrease in the levels of hs-CRP in moderately exercised older adults compared to baseline and nonexercise control values. The change in the level of hs-CRP significantly correlated with physical activity status and the improvement of cognitive abilities especially motor praxis, vasomotor organization, thinking operations, and attention and concentration in all participants. Many research studies reported multiple positive effects such as lower levels of hs-CRP [79, 80] and a better quality of life [81] among older adults participating in physical exercise training. Previously, higher serum levels of hs-CRP were shown to be closely related to cognitive disorders among older ages of both genders [82]. The positive effects of exercise training on the levels of hs-CRP depend on the mechanisms and type of exercise training [83, 84], which exerts a reduction in serum hs-CRP concentrations via decreasing associated metabolic risk factors [85], and changes in anthropometric parameters [86], of the participants.

Finally, the data obtained showed that physical activity status, inflammatory status, and oxidative stress played a pivotal role on cognitive performance of healthy older adults.

In conclusion, the data concluded that supervised moderate aerobic training for 24 weeks has a positive significant effect in improving cognitive functions via modulating redox and inflammatory status of older adults.

## Figures and Tables

**Table 1:** General characteristics of subjects.

Parameters	Control group (*n* = 50)	Exercise group (*n* = 50)
Male/female	30/20	35/15
Age (years)	67.3 ± 2.8	66.8 ± 3.7
BMI (kg/m^2^)	22.3 ± 2.7	23.5 ± 1.7^*∗*^
Waist (cm)	75.3 ± 10.2	86.3 ± 11.7
Hips (cm)	88.5 ± 5.2	87.5 ± 18.3
WHR	0.82 ± 0.07	0.98 ± 0.10^*∗*^
Systolic BP (mmHg)	122.2 ± 6.5	118.5 ± 10.8
Diastolic BP (mmHg)	78.5 ± 11.9	82.5 ± 10.3
Fasting blood sugar (mg/dL)	98.5 ± 6.3	105 ± 3.5
HbA1c (%)	6.2 ± 1.5	6.4 ± 1.9
VO_2_ max (mL/kg*∗*min)	32.6 ± 3.7	35.4 ± 2.9
Mean LOTCA score (SD)	97.8 ± 7.91	86.6 ± 8.24^*∗∗*^
LTPA (MET-H/week )	123.9 ± 15.6	96 ± 9.7^*∗∗*^

Values are expressed as mean ± SD; ^*∗*^ *P* < 0.05 and ^*∗∗*^ *P* < 0.01. Significance at *P* < 0.05.

**Table 2:** LOTCA scores in studied subjects following 12-week supervised aerobic training program (means ± SD).

Parameters	Control group		Exercise group	
	Pre	Post	Pre	Post
Orientation	12.8 ± 1.8	16.7 ± 2.5^*∗*^	9.0 ± 2.3	21.8 ± 0.5^*∗∗*^
Visual perception	18.2 ± 2.9	21 ± 0.98^*∗*^	11.3 ± 2.5	18.1 ± 1.9^*∗∗*^
Spatial perception	10.5 ± 0.4	13.5 ± 0.4^*∗*^	9.5 ± 2.1	21.13 ± 0.91^*∗∗*^
Motor praxis	8.8 ± 0.68	11.9 ± 0.52^*∗*^	9.4 ± 3.8	25.7 ± 2.3^*∗∗*^
Vasomotor organization	21.3 ± 2.6	25.1 ± 2.86^*∗*^	11.9 ± 3.7	38.1 ± 2.9^*∗∗*^
Thinking operations	23.7 ± 3.7	26.8 ± 2.95^*∗*^	9.6 ± 2.65	315 ± 2.6^*∗∗*^
Attention and concentration	3.7 ± 0.51	3.9 ± 0.18^*∗*^	2.1 ± 0.31	5.3 ± 0.45^*∗∗*^
Total LOTCA score	92.8 ± 6.3	98.9 ± 7.5^*∗*^	88.7 ± 8.3	110.8 ± 5.6^*∗∗*^

Values are expressed as mean ± SD; ^*∗*^ *P* < 0.05 and ^*∗∗*^ *P* < 0.01. Significance at *P* < 0.05.

**Table 3:** Changes in the level of oxidative stress and inflammatory markers and leisure-time physical activity (LTPA) score of participants following 12-week supervised aerobic training program (means ± SD).

Parameters	Control group (*n* = 50)		Exercise group (*n* = 100)	
	Pre	Post	Pre	Post
TAC (nmol/*μ*L)	16.5 ± 6.3	22.9 ± 11.7^*∗*^	9.7 ± 2.3	31.2 ± 5.1^*∗∗*^
MDA (*μ*mol/L)	4.7 ± 3.5	2.7 ± 1.7^*∗*^	15.5 ± 6.7	5.1 ± 1.8^*∗∗*^
8-OHdG (ng/mL)	0.98 ± 0.05	0.65 ± 0.08^*∗*^	25.7 ± 4.5	4.6 ± 2.9^*∗∗*^
hs-CRP (mg/L)	2.6 ± 2.1	1.8 ± 0.98^*∗*^	6.8 ± 2.5	2.8 ± 1.3^*∗∗*^
LTPA (MET-H/week)	123.9 ± 15.6	145.3 ± 14.5^*∗*^	96 ± 9.7	315 ± 17.6^*∗∗*^

Values are expressed as mean ± SD; ^*∗*^ *P* < 0.05 and ^*∗∗*^ *P* < 0.01. Significance at *P* < 0.05.

**Table 4:** Correlation coefficients analysis of oxidative damage and antioxidant biomarkers in relation to normal and abnormal hs-CRP levels.

Parameters	Normal (hs-CRP < 3.0) (*R*) (*n* = 50)	Abnormal (hs-CRP > 3.0) (*R*) (*n* = 50)
TAC (nmol/*μ*L)	−0.215^*∗∗*^	−0.157^*∗∗*^
MDA (*μ*mol/L)	0.245^*∗∗*^	0.235^*∗∗*^
8-OHdG (ng/mL)	0.512^*∗∗*^	0.540^*∗∗*^

Data presented as coefficient (*R*); ^*∗∗*^significance at <0.001.

**Table 5:** Posttraining correlation analysis of oxidative stress and inflammatory markers and cognitive abilities (LOTCA scores) variables according to the level of leisure-time physical activity (LPTA-MET-H/week) after 12 weeks of exercise.

Parameters	Exercise group (*n* = 100) (*R*)	
	(Low LTPA)	(High LTPA)
Total LOTCA score	0.571^*∗*^	0.561^*∗∗*^
Orientation	0.25 ^*∗*^	0.45^*∗∗*^
Visual perception	0.48^*∗*^	0.88^*∗∗*^
Spatial perception	0.651^*∗*^	0.263^*∗∗*^
Motor praxis	0.21^*∗*^	0.13^*∗∗*^
Vasomotor organization	0.421^*∗*^	0.425^*∗∗*^
Thinking operations	0.35^*∗*^	0.22^*∗∗*^
Attention and concentration	0.517^*∗*^	0.275^*∗∗*^
TAC (nmol/*μ*L)	0.429^*∗*^	0.312^*∗∗*^
MDA (*μ*mol/L)	−0.545^*∗*^	−0.668^*∗∗*^
8-OHdG (ng/mL)	−0.320^*∗*^	−0.541^*∗∗*^
hs-CRP (mg/L)	−0.350^*∗*^	−0.515^*∗∗*^

Data presented as coefficient (*R*); ^*∗*^significance at <0.01; ^*∗∗*^significance at <0.001.

**Table 6:** Posttraining correlation coefficients among factors involved in oxidative and inflammatory status and cognitive parameters (*n* = 100).

Parameters	TAC (nmol/*μ*L)	MDA (*μ*mol/L)	8-OHdG (ng/mL)	hs-CRP (mg/L)
Orientation	0.315^*∗∗*^	−0.450^*∗∗*^	−0.225^*∗∗*^	−0.610^*∗∗*^
Visual perception	0.215^*∗∗*^	−0.314^*∗∗*^	−0.250^*∗∗*^	−0.435^*∗∗*^
Spatial perception	0.198^*∗∗*^	−0.289^*∗∗*^	−0.365^*∗∗*^	−0.580^*∗∗*^
Motor praxis	0.311^*∗∗*^	−0.352^*∗∗*^	−0.465^*∗∗*^	−0.674^*∗∗*^
Vasomotor organization	0.250^*∗∗*^	−0.412^*∗∗*^	−0.512^*∗∗*^	−0.750^*∗∗*^
Thinking operations	0.413^*∗∗*^	−0.387^*∗∗*^	−0.535^*∗∗*^	−0.752^*∗∗*^
Attention and concentration	0.365^*∗∗*^	−0.478^*∗∗*^	−0.620^*∗∗*^	−0.588^*∗∗*^

Data presented as coefficient (*R*); ^*∗∗*^significance at <0.001.
